# Novel Microscopic Techniques for Podocyte Research

**DOI:** 10.3389/fendo.2018.00379

**Published:** 2018-07-11

**Authors:** Florian Siegerist, Karlhans Endlich, Nicole Endlich

**Affiliations:** Institute for Anatomy and Cell Biology, University Medicine Greifswald, Greifswald, Germany

**Keywords:** podocyte nephropathy, superresolution microscopy, multiphoton imaging, structured illumination microscopy, STED microscopy, atomic force microscopy, light-sheet imaging, serial block-face scanning electron microscopy (SBFSEM)

## Abstract

Together with endothelial cells and the glomerular basement membrane, podocytes form the size-specific filtration barrier of the glomerulus with their interdigitating foot processes. Since glomerulopathies are associated with so-called foot process effacement—a severe change of well-formed foot processes into flat and broadened processes—visualization of the three-dimensional podocyte morphology is a crucial part for diagnosis of nephrotic diseases. However, interdigitating podocyte foot processes are too narrow to be resolved by classic light microscopy due to *Ernst Abbe's* law making electron microscopy necessary. Although three dimensional electron microscopy approaches like serial block face and focused ion beam scanning electron microscopy and electron tomography allow volumetric reconstruction of podocytes, these techniques are very time-consuming and too specialized for routine use or screening purposes. During the last few years, different super-resolution microscopic techniques were developed to overcome the optical resolution limit enabling new insights into podocyte morphology. Super-resolution microscopy approaches like three dimensional structured illumination microscopy (3D-SIM), stimulated emission depletion microscopy (STED) and localization microscopy [stochastic optical reconstruction microscopy (STORM), photoactivated localization microscopy (PALM)] reach resolutions down to 80–20 nm and can be used to image and further quantify podocyte foot process morphology. Furthermore, *in vivo* imaging of podocytes is essential to study the behavior of these cells *in situ*. Therefore, multiphoton laser microscopy was a breakthrough for *in vivo* studies of podocytes in transgenic animal models like rodents and zebrafish larvae because it allows imaging structures up to several hundred micrometer in depth within the tissue. Additionally, along with multiphoton microscopy, lightsheet microscopy is currently used to visualize larger tissue volumes and therefore image complete glomeruli in their native tissue context. Alongside plain visualization of cellular structures, atomic force microscopy has been used to study the change of mechanical properties of podocytes in diseased states which has been shown to be a culprit in podocyte maintenance. This review discusses recent advances in the field of microscopic imaging and demonstrates their currently used and other possible applications for podocyte research.

## Introduction

Since podocytes are an essential part of the glomerular filtration barrier and any changes of the complex three-dimensional (3D) podocyte foot process morphology are associated with impairment or ultimately with a loss of kidney function, microscopic imaging of this specific cell is a fundamental technique for basic research as well as for pathohistological analysis for diagnosis of nephrotic diseases in the clinic. In the past, the ultrastructure of podocytes could only be visualized by transmission electron microscopy (TEM) as well as scanning electron microscopy, however these techniques are time-consuming and the preparation rather challenging.

Podocytes are specialized epithelial cells of the glomerulus and their function is highly dependent on their specific morphology. In contrast to the major processes of podocytes expressing predominantly microtubules, the podocyte foot processes are highly dependent on an intact actin cytoskeleton (1). Changes of the actin cytoskeleton by knockout, knockdown or mutations of actin-binding proteins have been shown to affect the morphology of the foot processes as well as the slit diaphragm which spans between the neighboring and interdigitating foot processes. This finally results in a so-called foot process effacement which is observed in patients suffering from nephrotic syndrome due to for example minimal change disease (MCD) (2) or focal and segmental glomerulosclerosis (FSGS) (3). Such foot process effacement leads to a loss of the glomerular permselectivity as well as to proteinuria, the excretion of a large quantity of high-molecular weight protein (4).

In this context, light microscopic imaging of podocytes *in situ* has been quite challenging because the size of foot processes and of the slit diaphragm lies below the optical resolution limit as defined by *Ernst Abbe*. This limit is dependent on the wavelength λ of the incident light divided by two times the numerical aperture of the objective used (5). For visible light and the usually used objectives in light microscopy, the maximum of the optical resolution is ~200 nm.

Therefore, techniques like scanning or transmission electron microscopy have been extensively used to study the morphology of the glomerular filtration barrier in health and disease (6). However, due to their complex 3D architecture, podocyte morphology has been challenging using standard TEM techniques due to the fact that physical sectioning produces geometric bias (7, 8). Although it was known that such TEM measurements are highly biased, they have been widely used as a standard procedure in podocyte research and renal glomerular pathology because of the absence of alternative techniques so far. Beside new upcoming strategies that enable electron microscopy for the spatial analysis of renal tissue, super-resolution fluorescence microscopy (SRM) overcoming *Ernst Abbe's* limit has found its way into kidney research during the last years. With multiphoton microscopy (MPM) as well as lightsheet/selective plane illumination microscopy (SPIM) fluorescence microscopy methods have been further developed making intravital imaging of podocytes in rodent and zebrafish possible.

Besides the huge advantages in the optic imaging systems, sample preparation has been also improved in the last years. Thus, with the new technique of expansion microscopy, it is possible to get 70 nm resolution images of cells and intact tissues using conventional diffraction-limited microscopes.

Beside this, modern confocal laser scanning microscopy-based methods like Airyscan (Zeiss) and HyVolution (Leica) microscopy are also available, however these specific microscopic techniques are not included in this review, although they are also used for basic renal research (9, 10). The following is focused on MPM, 3D-SIM, STED, dSTORM/STORM, PALM, 3D-EM, light-sheet, and atomic force microscopy (AFM).

## Multiphoton microscopy (MPM)

An issue for podocyte research is that tissue fixation and further processing causes numerous artifacts (shrinking, damage of cellular membranes, and cell organelles) which makes it hard to draw conclusions on the *in vivo* situation of the glomerular filtration barrier. Furthermore, it is not clear whether podocytes have been replenished after induction of podocyte injury or podocyte depletion by extraglomerular sources. Therefore, intravital long-term microscopy has to be used to directly track changes in real-time.

Although classic wide field (WF) microscopy has been used to study renal vascular physiology *in vivo*, e.g., in the hydronephrotic rat kidney (11) or to follow tubular reabsorption of fluorescent albumin in a model of direct podocyte damage in single rat nephrons (12), only indirect effects on podocyte function were visualized. Besides this, confocal laser scanning microscopy has been used to study isolated, genetically labeled fluorescent glomeruli *ex vivo* using a podocyte-specific dual color (13) or confetti reporter approach (14). Unfortunately, these imaging strategies are clearly limited in their feasibility to resolve individual podocytes *in vivo* due to the small ability of tissue penetration of the incident light.

**Figure 1:**
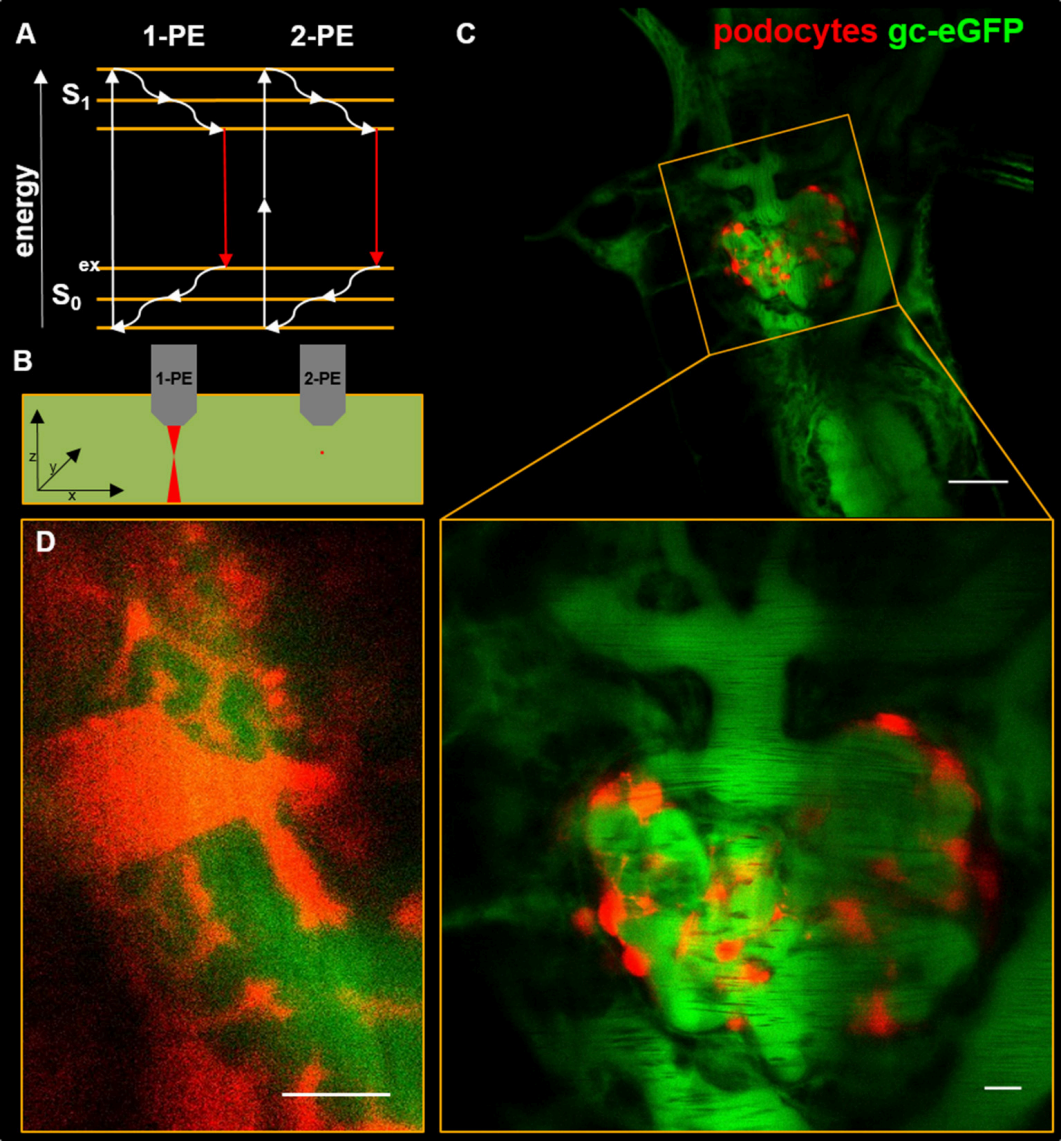
The Jablonski diagram in **(A)** shows the energy levels of fluorophore electrons upon single-photon excitation (1-PE) and two-photon excitation (2-PE). For fluorescence emission, electrons are elevated from the S_0_ to the S_1_ level where they fall back to S_0_ while emitting a photon. Dense temporal concentration of exciting photons of longer wavelengths compared with 1-PE elevates fluorophores up on the S_1_ level with subsequent emission of a photon. **(B)** shows a scheme of 1-PE and 2-PE in a homogenously fluorescent liquid. Compared to 1-PE the fluorescence emission (red) of 2-PE excitation is spatially restricted to a small point within the focal plane. **(C)** shows an *in vivo* multiphoton micrograph of a transgenic zebrafish larva (5 days past fertilization) with expression of mCherry in podocytes and 78 kDa gc-eGFP fusion protein in the blood plasma. The magnification in **(D)** shows interdigitating major processes on the face of the glomerular capillaries. The scale bars indicate 50 μm in **(C)**, 10 μm in the magnification of **(C)** and 5 μm in **(D)**.

Already in the year 1931, the new concept of two-photon excitation was described by Goeppert-Mayer which was applied by Denk and colleagues to accomplish deeper tissue penetration (15). As shown in Figure 1A, two-photon excitation uses the physical effect, that in the case of tight temporal concentration of exciting photons, two photons with about double of the excitation wavelength that is necessary to excite the fluorophore in the classical one-photon excitation way, can be used to transfer the electron into the excited level S_1_. From this excited state, the electron drops down to the ground state S_0_ by emitting one photon. For example, eGFP which is normally excited by a wavelength of 488 nm, an excitation wavelength of around 900 nm is necessary. Therefore, two-photon excitation wavelength has to be determined for every fluorophore used (16).

Commercially available systems accomplish that by using femtosecond pulsed Ti-Sapphire lasers working within the near-infrared spectrum. Due to the effect of two-photon excitation and longer excitation wavelengths, fluorescence emission is spatially restricted to the focal point significantly decreasing phototoxicity and photobleaching to a minimum. Compared to conventional fluorescence microscopy systems, this spatially restricted fluorescence emission leads to a dramatic decrease of out-of-focus fluorescence, which in single-photon laser scanning systems needs to be filtered out by a pinhole to enable optical sectioning and increase spatial resolution (Figure 1A). With this new technique, it is possible to penetrate the kidney tissue up to several hundred micrometers in depth.

As maximum fluorescence excitation is spatially restricted and highly focused to a single point within the focal plane, multiphoton-excitation has also been used to manipulate cells *in vivo* parallel to microscopic imaging. Different working groups showed that MPM can be used to precisely damage subcellular structures (17), perform single-neuron axotomies (18), manipulate neurons (19), and either puncture or occlude small blood vessels in different stroke models (20).

Additionally to *in vivo* imaging purposes, MPM is an appropriate option if large z-stacks of tissue sections and a higher spatial resolution compared to lightsheet systems are needed. Recent work by Puelles and colleagues combined MPM with optical clearing of kidney tissue to quantify podocyte number and size simultaneously in the whole kidney of mice. Using this approach MPM can be applied to efficiently quantify podocyte depletion in different disease models (21–23).

In 2002, the group of Molitoris applied MPM to image renal tubules, vasculature and glomeruli in exteriorized kidneys of anesthetized rats. To label structures of interest, fluorescent dyes were injected into vessels for the labeling of nuclei (e.g., Hoechst 33342), apoptotic and necrotic cells (propidium iodide), the vasculature (high molecular weight dextran), and to demonstrate glomerular filtration by the appearance of Texas Red-labeled gentamycin within proximal tubule cells (24).

The first researchers using MPM to image glomerular morphology were Peti-Peterdi and colleagues who microscopically imaged the juxtaglomerular apparatus of isolated, perfused glomeruli. Additionally, MPM imaging was combined with the ability to measure changes of intracellular Ca^2+^ concentration non-invasively and selectively image fluorescence-labeled renin granules at the afferent arteriole (25–27).

Following their first pioneering work, Molitoris and colleagues demonstrated that MPM could be applied to measure glomerular sieving coefficients of intravenously administered fluorescence-labeled albumin into rats (28). In this work, the group showed that in healthy animals, nephrotic levels of albumin with a glomerular sieving coefficient of ~0.034 passed the glomerular filtration barrier. Challenged by the fact that only minimal amounts of albumin are found in the urine of healthy specimen, the group hypothesized that filtered protein subsequently had to be taken up by proximal tubule cells. Therefore, the researcher concluded that the occurrence of nephrotic range proteinuria was independent of glomerular filtration barrier function and solely explained by impaired endocytosis of macromolecules by proximal tubule cells (29). However, those paradigm-shifting findings were challenged by subsequent work of two independent researchers using the same strategy. Thus, Tanner and Peti-Peterdi found significantly lower values for the glomerular sieving coefficient of albumin (0.002–0.004) dependent on the microscope they used (30, 31). This is in agreement with previous findings obtained from micropuncture studies. Later, these findings were confirmed by Nakano and colleagues in mice (32) and by Schießl and Castrop in healthy rats highlighting the role of the glomerular filtration barrier (33, 34). It is most likely, that false-high values were produced due to illumination of the detector by scattered out-of-focus fluorescence in external detector systems and suppressed in internal detection setups (30, 35).

In the following years, Peti-Peterdi and colleagues used intravital MPM in rats to image podocytes with the help of intravascular administration of freely filterable but membrane-impermeable dyes like Lucifer yellow. Due to the appearance of these dyes within Bowman's space, podocytes were negatively labeled as non-fluorescent structures within the fluorescent primary urine (36). In this work, highly migratory cells were shown at glomerular capillaries which were considered to be podocytes. Additionally, sub-podocyte pseudocysts, meaning dilatations of the sub-podocyte spaces were described in a PAN-induced nephrosis model. Later, these spaces have further been characterized by Schießl and colleagues in rats demonstrating transcytosis of material from the sub-podocyte space into Bowman's capsule (34, 37). Later, Burford et al. used MPM in transgene mice expressing a fluorescent GCaMP3 as an endogenous Ca^2+^ indicator showing cell-to-cell propagating Ca^2+^ waves along the glomerular tuft following focal podocyte damage using the focused laser beam (38).

In 2012, Khoury and co-workers used MPM in a transgenic, GFP-expressing mouse strain to visualize individual podocytes in freshly explanted kidneys (39). Interestingly, they observed that female mice have more sub-capsular glomeruli than male making them more feasible for intravital imaging.

In a work by Hackl and colleagues, podocytes were specifically labeled using endogenous expression of fluorescence proteins using a confetti-reporter approach (40). In these mice, individual podocytes could be imaged multiple times over several days and single podocytes were identified to migrate to the parietal layer of Bowman's capsule in a model of unilateral ureteral ligation. Additionally, the formation of so-called nanotubes between podocytes and parietal epithelial cells was observed. However, this controversial finding of migrating podocytes was later challenged by intravital MPM studies of fluorescence protein-labeled podocytes in transgene mouse models by Brähler et al. (41). In this work, dynamic apical membrane protrusions were visualized in two independent injury models (constitutive active Rac-1 expression and nephrotoxic serum nephritis) but no lateral migration along the glomerular basement membrane (GBM) was observed.

Using different transgenic fluorophore-expressing mice, the response of glomerular, cells of renin-lineage have been tracked in a podocyte-depletion (42) and a mesangiolytic model (43) both showing migration of these juxtaglomerular cells to the glomerular tuft.

Unfortunately, a pitfall of MPM in living rodent is that only superficial, sub-capsular glomeruli are accessible for MPM-imaging as tissue penetration in the optical dense kidneys lies in the range of 100 μm. Therefore, rodent strains that possess larger numbers of superficial glomeruli like the Munich-Wistar-Frömter rat (44) or BALB/c and C57BL/6 mice (45) have been utilized for efficient *in vivo* imaging.

Along other longer established models like mice or rats, larval zebrafish offer several advantages especially for *in vivo* imaging of podocytes. Three days after fertilization, zebrafish larvae possess a filtering glomerulus attached to a pair of renal tubules with high structural and genetic analogy (46–49). Using MPM of transgene, eGFP-labeled podocytes, our group visualized podocyte precursors migrating to the midline and differentiating into podocytes (50). Additionally, we showed that in healthy zebrafish larvae, podocytes were stationary cells, neither showing lateral migration nor a change of the branching pattern of interdigitating major processes (50).

Since we have shown that MPM can be used to visualize healthy podocytes in zebrafish larvae, we used this approach to investigate morphological changes of podocytes as well as dilatations of Bowman's space after morpholino-induced gene knockdown (51, 52).

Beside this, our group used a selective transgene podocyte injury model established by Zhou and our group (53–55) in which administration of metronidazole leads to specific ablation of podocytes in a transparent zebrafish strain. Since the podocytes of this specific zebrafish strain expresses the fluorescence dye mCherry, *in vivo* imaging is possible (55). By breeding of this established transgenic strain with the zebrafish strain expressing the eGFP-labeled vitamin D-binding protein in the vasculature (Figure 1B), we were able to study changes of the glomerular filtration barrier due to podocyte injury (54) as well as of the podocyte morphology (e.g., major processes, Figure 1C) (53) directly *in vivo* by MPM. Recently, we demonstrated that after an induction of podocyte injury, podocytes show massive foot process effacement, sub-podocyte space pseudocyst formation and detachment of podocyte clusters from the GBM that resemble changes observed in rodents and biopsies of patients suffering from kidney diseases (56, 57). Furthermore, we were able to show that no lateral migration of podocytes or immigration of extra-glomerular cells occurred during the early phase of podocyte injury (53). However, these current findings are restricted to pronephric podocytes in larval zebrafish and the situation in the adult mesonephric kidney remains unclear.

Another tempting application of MPM is the intravital and label-free fluorescence imaging. Herein, tissue autofluorescence for example deriving from the reduced form of nicotinamide adenine dinucleotide (NADH) can be used to image native tissue without additional transgenic or chemical labeling (58, 59). Additionally, label-free microscopy has been combined with fluorescence life time imaging (FLIM) which basically instead of giving fluorescence intensities, gives excited-state decay rates for every pixel (58). In the kidney field, Hato and colleagues used intravital MPM combined with FLIM to show characteristic FLIM profiles of different tubular segments depending on the individual metabolism. Additionally, the researchers showed that in contrast to several tubular segments, two-photon excited autofluorescence was not bright enough to image podocytes in rats presumably due to relatively low endogenous NADH levels which was overcome using FLIM (60).

## Selective plane illumination/light-sheet microscopy

Additional to the previous described concepts to perform optical sections of biological specimen using either a pinhole in C-LSM or focal point restricted fluorescence emission in MPM, another concept has been developed in 2004. Huisken and coworkers presented a method named selective plane illumination microscopy (SPIM) or light-sheet microscopy (61). In this technique, a single plane in a thick specimen like a living embryo or fixed tissue is two-dimensionally illuminated by a thin light-sheet. Fluorescence emission is visualized by a WF detection system orthogonal to the illumination. Through serial movement of the light-sheet within the volume of the sample, it is therefore possible to perform optical sections over a range of up to several millimeters. Due to these advantages, and combined with the high temporal resolution, light-sheet microscopy has been widely used, especially in developmental biology (62).

In 2017, Klingberg and colleagues developed an approach to quantify total glomerular number combined with capillary tuft size in complete murine kidneys (63). To achieve this, the researchers developed a specialized protocol combining tissue clearing and immunolabeling of CD31^+^ endothelial cells. In a murine model of nephrotoxic serum (NTS) nephritis the authors found that compared to control animals, total glomerular number significantly declined by about 5,000 glomeruli per kidney after 14 days. Using this method, the authors found a significant increase in glomerular tuft volume in NTS-treated mice. Furthermore, this technique allowed to compare average single-nephron creatinine clearance between different animals. Held and colleagues presented a method, in which self-assembled kidney organoids grown from murine embryonic kidney tissue were imaged using light-sheet microscopy (64). For this method, the authors took advantage of the CLARITY method (65) to clear tissue for effective deep volume imaging. In 2017, Isaacson and coworkers showed that light-sheet microscopy was not only feasible for the visualization of model organisms, but also for 3D imaging of a complete human fetal kidney stained for E-cadherin and HoxA11 (66).

## Super-resolution microscopy

In 2014, Stefan Hell, William Mourner, and Eric Betzig were awarded the Nobel Prize in chemistry for their achievements to overcome Abbe's limit using different strategies, summarized under the term super-resolution microscopy (SRM). Since the establishment of SRM, different types of microscope techniques like STED, 3D-SIM, (d)STORM, and PALM were used giving novel insights in morphology and behavior of cells. In the following, these techniques will be described in more detail.

### Three dimensional structured illumination microscopy (3D-SIM)

**Figure 2:**
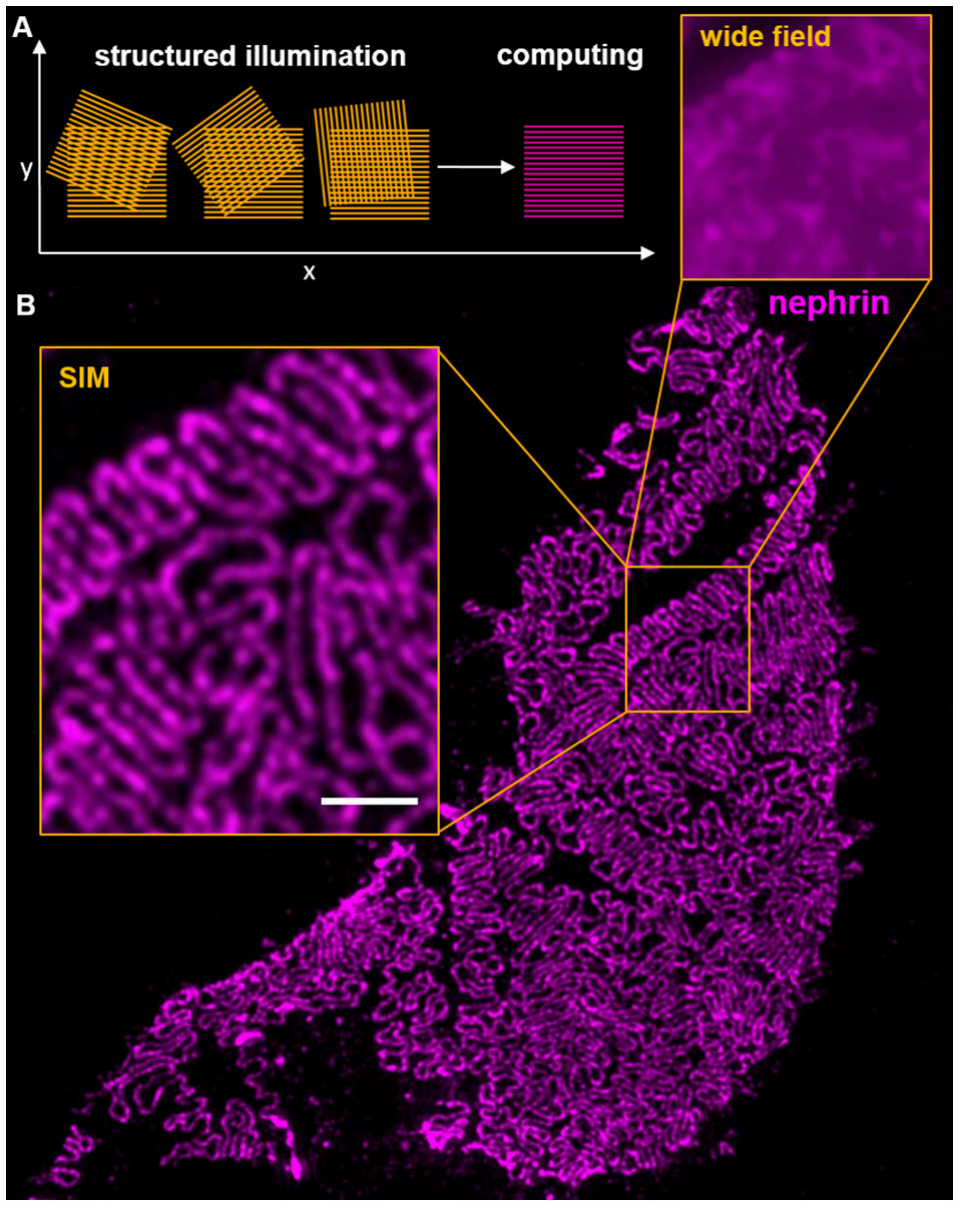
The scheme in **(A)** shows the interference of an illumination pattern in three different angles with the probe. In the first phase, vertical Moiré patterns are clearly visible and resolvable by widefield microscopy. **(B)** shows a histological section of a murine glomerular capillary stained for nephrin. The inserts show the gain of resolution compared from widefield microscopy and 3D-SIM. Clearly, individual interdigitating foot processes are resolvable on the glomerular capillary. The scale bar indicates 1 μm.

Three Dimensional Structured Illumination Microscopy (3D-SIM) uses illumination of a probe through a defined grating to demodulate high frequency patterns of a sample by interference of the grating with the probe. This interference results in so-called Moiré patterns with a lower spatial frequency compared to the original pattern of the probe (Figure 2A). Normally, three to five different angles and five phase shifts are used, so that 15–25 WF pictures are computed to one single SIM image. These Moiré patterns can be resolved by WF microscopy and subsequently back transformed by specialized software (67, 68). One main advantage of SIM is, that it can be used with a large variety of standard fluorophores, embedding media and conventional sample preparation (69). It therefore allows the re-evaluation of samples that were already imaged using conventional approaches leading to new insights in existing material.

A disadvantage of 3D-SIM is that compared with WF and confocal laser scanning microscopy it is rather a qualitative than a quantitative method. 3D-SIM allows powerful spatial analysis of expression patterns of proteins of interest with problems in quantitative comparison of fluorescence intensities, which in the meantime was partially solved by software applications (70). Using this technique, it is possible to achieve resolutions up to 85 nm in the x,y-dimension and 250–300 nm in the z-dimension, resulting in an almost 10-fold increased voxel resolution as compared to conventional methods.

Lately, 3D-SIM has been improved due to combination with an adaption of SPIM (Bessel beam plane illumination), which limits fluorescence emission to the vicinity of the focal plane (71). Therefore, phototoxicity and photobleaching is highly decreased enabling high-resolution imaging of sub-cellular dynamics combined with higher temporal resolution even in thicker biological samples and living cells (72). In 2014, Zhao and colleagues used this approach to visualize mCherry-labeled podocyte cell bodies and EGFP-labeled endothelial cells in larval zebrafish (73).

In 2016, Pullman and colleagues showed that 3D-SIM can be used to visualize podocyte foot processes using immunofluorescence staining of cryosections with FITC-labeled podocin antibodies (74).

As shown in Figure 2B, our group showed that using 3D-SIM with standard-processed formalin-fixed and paraffin-embedded tissue sections originating from the histopathological routine, podocyte foot processes could be visualized and morphometrically examined (8). Additionally, we established an open source, FIJI-based (75) semi-automatized software technique named *PEMP* (***P****odocyte* ***E****xact Morphology* ***M****easurement* ***P****rocedure*) to quantify changes of podocyte morphology as well as the slit diaphragm. For this purpose, we automatically segment and quantify the length of the slit diaphragm per glomerular capillary area of kidney sections that were stained with an antibody against the slit diaphragm protein nephrin. In a first proof-of-principle study, we showed that characteristic changes in patients diagnosed for MCN could be examined using *PEMP* and that the slit diaphragm density (SD) directly correlates with the foot process width. We further could show that, due to geometric bias, measurements of foot process width made in TEM pictures are significantly higher and showed a higher variance than 3D-SIM measurements of the same individual (8).

**Figure 3:**
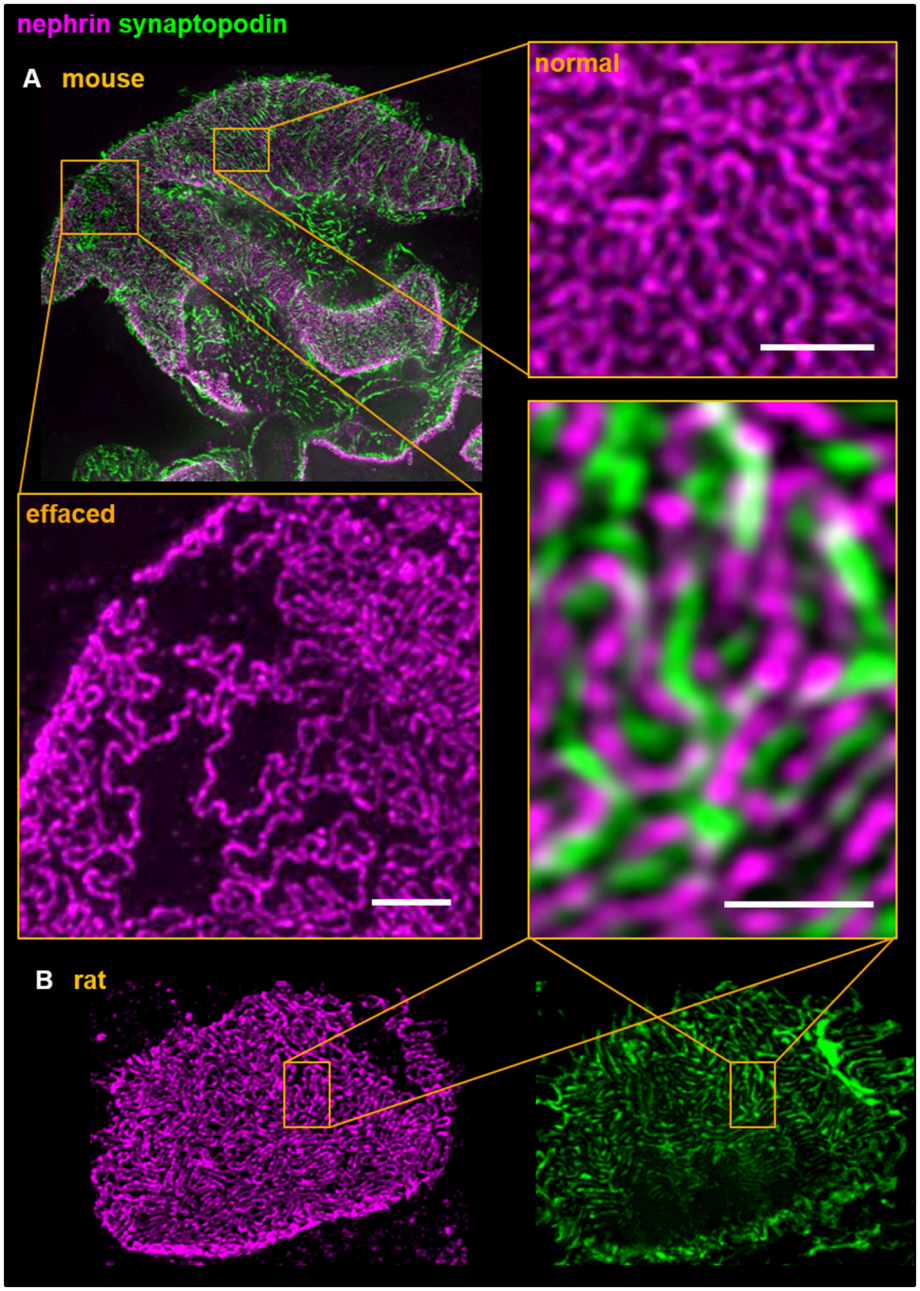
Dual color 3D-SIM of rodent **(A)** in mouse and **(B)** in rat) paraffin kidney sections shows the interdigitating foot process pattern of podocytes. Foot processes and the slit diaphragm were labeled using anti-synaptopodin and anti-nephrin antibodies, respectively. Although both proteins are in close structural vicinity the individual staining patterns do not overlap and therefore demonstrates the high resolution accomplished by 3D-SIM. All scale bars represent 1 μm.

Since there are a high numbers of rodent-based gene disruption models as well as drug-treated animals showing a renal phenotype like proteinuria, we extended our technique to mice and rats. As shown in Figure 3, we can further visualize rat and mouse foot processes by double staining for the slit diaphragm protein nephrin and for the actin-associated protein synaptopodin that is located in the center of the foot processes as shown by multicolor 3D-SIM. Figure 3A reveals areas with broadly effaced cellular protrusions lying in close vicinity to areas with healthy interdigitating foot processes in 3D-SIM images of mouse kidney sections indicating the heterogeneity of foot processes in glomeruli of healthy subjects. This underlines the need of an accurate and objective quantification of the foot processes and slit diaphragm density to determine the changes of the morphology of podocyte foot processes which could be used to compare and to follow the process of podocyte effacement in different animal models.

### Stimulated emission depletion microscopy (STED)

STED microscopy was theoretically developed by Hell and Wichmann in 1994 (76). Some years later, STED was technically applied and improved in a non-biological setting (77–79). To accomplish an increase of resolution, STED uses selective depletion of fluorescence emission around a focal point. Practically this is accomplished by an exciting central beam and a donut-shaped emission depletion beam in a laser scanning setup which forces electrons from the S_1_ to the S_0_ level without emission of a photon in the emission spectrum. Therefore, the point of fluorescence emission is significantly decreased and resolution is improved (76). Since then, STED is increasingly used in a variety of biological questions (80). Lately, STED microscopy has been further improved by using only one exciting, donut-shaped laser beam with a minimum-emission center. Therefore, the fluorophores residing in the center of the beam will be in the OFF-state upon illumination and the position of these individual non-emitting fluorophores is then calculated. Especially tempting about this concept named MINFLUX is, that the center of minimum excitation is dependent on photon fluxes in the surrounding beam. Therefore, the center can be adjusted so small, that only single fluorophores will be in the center leading to single-molecule microscopy in a scanning setup with optical resolutions under 10 nm (81).

The first STED microscopy study in a renal setting was conducted by Unnersjö-Jess and colleagues who resolved individual foot processes that were labeled with antibodies against the slit diaphragm proteins nephrin and podocin in optically cleared rat kidney tissue using STED microscopy (82). Additionally, they demonstrated that characteristic effacement of foot processes was resolvable in the passive Heymann nephritis model in rats. In a follow-up study, Unnersjö-Jess and colleagues combined STED microscopy with volumetric tissue expansion (see section about tissue expansion microscopy) and tissue clearing of murine kidney tissue to increase the resolution obtained (83).

### Localization microscopy: STORM, dSTORM, PALM, and DNA-PAINT

**Figure 4:**
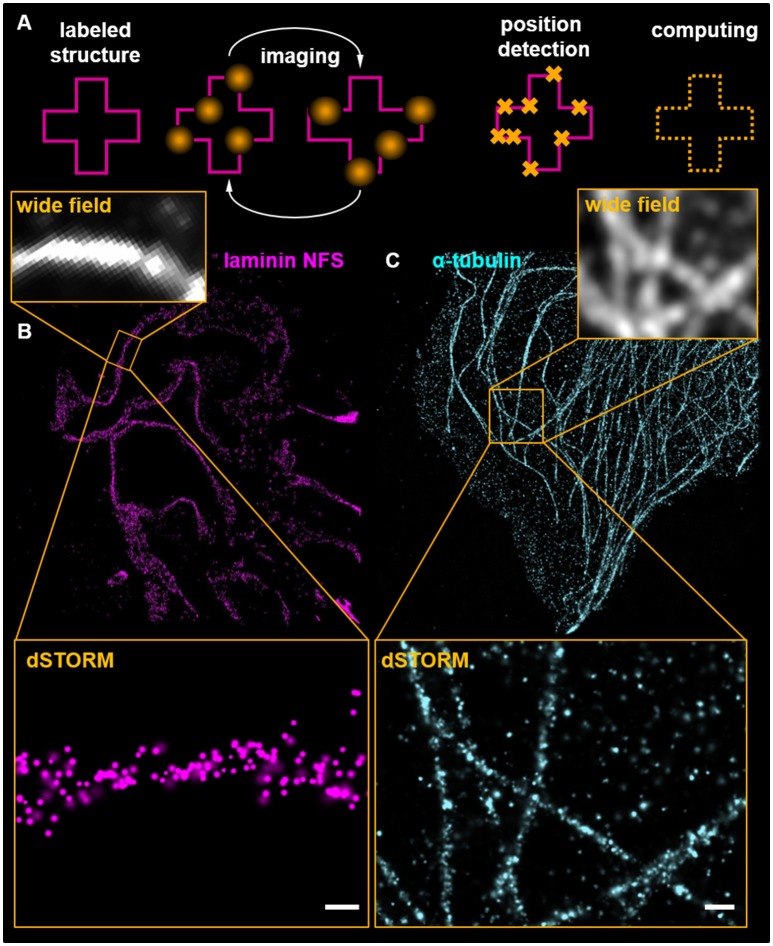
Scheme **(A)** presents the basic principle of localization microscopy. Labeled structures are cyclically imaged and positions of individual fluorophores calculated. For further explanations see the main text. **(B)** shows a dSTORM image of a murine glomerulus labeled with an antibody for the glomerular basement membrane component laminin detected by secondary antibodies conjugated to Alexa Fluor 647. Ten thousand imaging cycles were recorded in the presence of a redox buffering system containing 2-mercaptoethylamine and in absence of oxygen accomplished by glucose oxidase and catalase. Superresolution is demonstrated in comparison with the corresponding WF picture. The scale bar indicates 200 nm in the magnification. **(C)** shows the microtubule cytoskeleton of a murine cultured podocyte detected by anti α-tubulin antibodies. The magnification demonstrates localizations of individual antibodies on microtubules. The scale bar indicates 500 nm.

The SRM strategies compiled under the topic localization microscopy surpass the optical determined resolution limit by the detection of individual fluorophores using different strategies. The most frequently used techniques are photoactivated localization microscopy (PALM) (84), stochastic optical reconstruction microscopy (STORM) (85), direct STORM (dSTORM) (86), and DNA-PAINT (87, 88). All these techniques are based on the time-resolved imaging of ON/OFF cycles of fluorophores using different approaches. While DNA-PAINT uses temporary hybridization of fluorescence-labeled DNA oligonucleotides with their antisense-strand attached to primary antibodies (88), PALM and STORM/dSTORM require fluorescence proteins or organic dyes that can be exogenously switched. For PALM, a specific fluorescent protein photo-switches from one emission wavelength to another upon the excitation (89). As PALM requires the transgene expression of fluorescence proteins like *Kaede* or *Dronpa*, it has been mainly used in *in vitro* approaches like cell culture (90). In contrast to that, widely used cyanine dyes have the ability to switch between a bright ON- and a dark OFF-state following high-power illumination which is facilitated in the presence of a specific redox buffer system (91). As shown in Figure 4A, stochastically shifting between these ON/OFF-states can be observed as a blinking of the fluorophores.

All methods have in common, that during each imaging cycle only a sparse subset of fluorescence proteins are in the ON-state. Therefore, the detected fluorescence signal stochastically originates from single molecules. Detected by a pixelated detector, these ON-signals can be fitted to a centroid position of a point spread function determining the most probable localization of the fluorophore (85). In contrast to STED microscopy, in which the obtainable resolution is correlated with the intensity of the emission depletion ring, in localization-SRM, localization precision in inversely correlated with the emitting photon yield reaching the detector. Therefore, to increase localization precision, bright dyes like Alexa Fluor 647 and 488 have to be used. During an imaging experiment, a large number of frames (~10,000 frames) have to be recorded and analyzed by specific algorithms. Using this software, the position of each signal can be localized and evaluated (85, 92, 93). Since image acquisition needs a longer time period, accurate drift correction using fluorescent fiducial markers has to be performed. Additionally, in the case of multicolor localization microscopy, channels have to be carefully aligned to account for chromatic aberrations of the optical system.

Beside this, the qualities of antibodies used for STORM/dSTORM as well as careful sample preparation techniques are highly essential to generate SRM pictures. In case of live-cell approaches, techniques like SNAP-tags combining labeling of living structures with organic dyes by transgene fusion proteins can be used (94). A possible alternative to that are the development of single domain antibodies (or V_H_ antibodies) deriving from alpacas which can be fused to fluorophores and expressed in living cells or small organisms (95). However, imaging conditions like specific buffers have always to be fine-tuned for each imaging purpose (96).

In Figure 4B, a dSTORM image of the murine GBM component laminin is visualized. Figure 4B shows laminin that was labeled with a polyclonal antibody against the whole protein (not further specified: NFS). A higher magnification of the SR-image is shown in the insert of Figure 4B. The individual localizations show that the laminin NFS antibody binds mostly the GBM. Figure 4C shows a dSTORM image of the microtubule meshwork of a murine cultured podocyte. The increase of resolution is demonstrated in the WF microscopy inserts in Figures 4B,C.

In a first approach in 2013, Suleiman et al. used correlative STORM and scanning electron microscopy to map positions of proteins of the GBM using antibodies directed against various components like agrin, collagen IV, and laminin (97). In another study, in which they correlated STORM and scanning electron microscopy showing the localization of EGFP-Rac-1 in murine podocytes, they found that proteinuria and podocyte foot process effacement was rapidly induced after activation of EGFP-Rac (98). In 2017, this established technique was further used in a study conducted by Schell and colleagues to investigate the subcellular localization of the FERM protein EPB41L5. Triple color STORM revealed, that this protein localizes in close proximity to the integrin β1-labeled base of podocyte foot processes which were additionally stained with an anti-podocalyxin antibody (99). In a follow-up study, Suleiman and colleagues used STORM to investigate components of the podocyte slit diaphragm, actin cytoskeleton and actin-associated proteins like myosin IIA (10). Recently, STORM was used by the same group to show integration and correct orientation of human laminin 521 in the GBM in a murine model of Pierson-syndrome (*lamb2*^−/−^) following intravenous injection of laminin 521 (100).

## Expansion microscopy

Expansion microscopy (ExM) is a newly developed technique that generates super-resolution images on conventional and low-cost microscopes. For this purpose, biological samples are embedded in a matrix of swellable polymers, digested and physically expanded. Until today, different methods of ExM are used: In the first method as used by Unnersjö-Jess and colleagues combined with STED (83) (mentioned before in SRM), tissue is embedded in hydrogel, expanded and subsequently immunolabeled (101). In the second approach described by Chen et al. (102), sections are first immunolabeled with fluorophore-conjugated antibodies, and finally fluorophores are covalently fixed within a polymer meshwork in which the tissue is embedded. After digestion of proteins, the polymer is physically expanded and imaged by fluorescence light microscopy. Using this technique, it is possible to reach super-resolution using conventional diffraction limited microscopes (102). Moreover, it is possible even to overcome the resolution limits of SRM by the new approach named UltraExM that was recently shown by Gambarotto et al. (103). They were able to reveal the ultrastructural localization of tubulin in centrioles in high resolution details.

In 2017, an improved ExM protocol has been published which uses samples obtained from routine histopathologic assessment which was therefore named ExPath (104). Very promising about this approach is, that formalin-fixed paraffin embedded (FFPE) routine material which already had been stained with hematoxylin and eosin was rehydrated and used for subsequent immunolabeling and tissue expansion with a resulting expansion factor of around 4 leading to a lateral resolution of ~70 nm. Therefore, the possibility of using archived material for histopathologic assessment with following immunofluorescence ExM on the same slide is given. In the original ExPath work, the authors used their approach to demonstrate podocyte morphology in human histopathologic FFPE or fresh frozen renal biopsy samples probed with antibodies directed against synaptopodin and α-actinin (104). Herein, the authors demonstrated that using ExPath on FFPE sections had an accuracy of 90% to diagnose foot process effacement in ten cases of FSGS and MCD.

## Serial block face/focus ion beam scanning electron microscopy, electron (cryo)tomography

One of the first techniques which enabled volumetric electron microscopy imaging in dimensions necessary for imaging of the three-layered glomerular filtration barrier was serial block face scanning electron microscopy introduced by Denk et al. (105) and focused ion beam microscopy (106). Both techniques display a significant improvement of the methods used until then, in which serial sections had to be cut on an ultramicrotome, imaged and aligned afterwards (107). In both approaches, tissue is fixed, contrasted *en bloc* and either embedded in epoxy resin or deep frozen. Serial sections (~50 nm) of the tissue are either cut by an ultramicrotome or milled by a focused ion gun integrated in a scanning electron microscope. After every section, an electron micrograph is scanned of the face of the remaining tissue block. Subsequently, the resulting image stack is segmented for desired structures which then can be reconstructed in three dimensions with a voxel size of down to 3 × 3 × 3 nm. In 2013, an approach to image −150°C hydrated frozen, native tissue in a FIB-SEM setup has been made demonstrating a voxel size of 7.5 × 7.5 × 30 nm over a z-distance of 3.81 μm in an optic nerve (108). Herein, endogenous contrast was sufficient to track cellular organelles over the imaging distance without additional contrasting.

In 2014, Arkill and colleagues presented the first work that used FIB-SEM and SBF-SEM to visualize the 3-D architecture of the glomerular filtration barrier using additional special fixation demonstrating the glomerular glycocalyx (109). In studies of 2015, Ichimura and colleagues as well as Burghardt and colleagues used SBF-SEM and/or FIB-SEM to demonstrate morphology of rat and murine podocytes *in situ* (110, 111). In this study, the authors showed that major processes are connected to the GBM by ridge-like prominences (111). In a follow up study, Ichimura and colleagues investigated structural changes in the developing rat glomerulus showing the different morphological alterations during nephrogenesis (112). Randles and coworkers used SBF-SEM to reconstruct podocytes of healthy mice and of mice models with Alport syndrome (113). In 2017, Lausecker and colleagues applied SBF-SEM to three-dimensionally reconstruct interdigitating podocytes on the GBM and to compare podocyte morphometry between control and podocyte-specific vinculin knockout mice (114).

Another groundbreaking addition to the electron microscopy toolbox was the development of electron tomography (ET) and electron cryotomography (cryoET). Basically, tomography facilitates three dimensional reconstruction of an object from a series of two-dimensional projections as first theoretically described by Radon (115). In ET, two-dimensional projections are recorded through serial tilting of a sample in a TEM setup. This produces a series of two-dimensional images in which the spatial information is than digitally reconstructed using 3D tomography. A prerequisite for efficient ET is to keep the morphology of the sample in almost the native structure. This was achieved through embedding the sample in epoxy resin and further enhanced when a procedure named vitrification was developed that could preserve structure up to the level of protein complexes. This is achieved by rapid freezing so that water molecules do not arrange in an ordered crystalline fashion instead forming an amorphous matter named vitreous ice. There are basically two methods to achieve this. For small samples like isolated proteins or individual cells vitrification is achieved by immersion in liquid ethane whereas tissue sections are vitrified by high pressure freezing (116).

A limiting factor for high resolution in ET is a required sample size of <500 nm so that electrons can still pass the sample, but the sections also contains enough spatial information to answer the question of interest. To achieve this, different strategies are applied: The first is to mill the samples within a cryoET using a FIB until the desired thickness is achieved. The second is that the sample after vitrification is trimmed by cryo-ultramicrotomy (117). Using this technique, it is not only possible to image cells and tissue, but also individual macromolecules in their near-native state.

Already in 2004, after Nephrin was identified to be mutated in steroid resistant nephrotic syndrome of the Finish type and to localize specifically to the slit diaphragm (118), Wartiovaara and colleagues used ET of epoxy-resin embedded kidney samples and cryoET both combined with immunolabeling in human, mouse and rat tissue to unravel the structure of the slit diaphragm. As the opposing nephrin strands formed homodimers with varying gaps with a smaller size than an individual albumin molecule, the researchers concluded that the slit diaphragm can provide a physical barrier to plasma macromolecules. Additionally, the authors described an occasional local horizontal stratification of the slit diaphragm. Additionally to the aforementioned FIB-SEM data, Burghardt and colleagues presented also ET data of murine and human samples demonstrating the previously described two-layered structure of the slit diaphragm in neighboring foot processes (110). In 2013, Cheng et al. demonstrated ET on murine fixed kidney tissue. For 3D reconstruction by ET, the researchers used 270 nm epoxy resin semithin sections and showed 3D morphology of glomerular endothelial cells, the GBM and podocytes (119).

In 2016, Grahammer and colleagues used cryoET to image the interdigitating pattern of individual nephrin but now also neph1 homodimers in murine podocyte foot processes in wild type as well as in nephrin and neph1 knockout mice (120). In this study, the researchers showed that the bipartite slit diaphragm is composed of the two integral components nephrin and neph1 which form two ordered layers within the slit diaphragm with an apical nephrin and a basal neph1 layer. Additionally, they performed *in silico* analysis of the nephrin and neph1 showing that the Ig folds of both proteins are rather flexible. Given this flexible behavior of these proteins, the authors of the study concluded that contrary to earlier interpretation, the slit diaphragm does not form a physical barrier to molecules traversing the GBM (120).

## Atomic force microscopy

In 1986, Binnig and Quate presented a first proposal for a microscopic technique they named atomic force microscopy (AFM) (121). In the first setup, a small tip was mounted on a flexible cantilever which directly interacted with a sample (contact mode) scanning its surface. The position of the tip can then be tracked by a laser that is being deflected upon movement of the cantilever. However, for biological samples this mode is rather unpractical as most samples are in danger of being destroyed upon direct contact. In 1987, Martin et al. overcame this limitation as they modified AFM in the way that the cantilever oscillated in high frequency with the tip in close proximity to the sample surface without direct interaction (122). By measuring forces used to impress cellular surfaces, AFM is capable to combine functional studies of mechanical property of cells with morphologic data of cellular surfaces (123).

As tensile stress in form of circumferential wall tension as well as fluid shear stress has been shown to play an important role in podocytopathies, evaluation of mechanical properties of these cells and their response to external mechanical stressing still is a topic of interest (124). In 2010, Welsh and colleagues applied AFM to visualize the cellular surface of cultured podocytes upon application of insulin. Herein they showed, that human immortalized, cultured podocytes rapidly changed their phenotype and retracted their cellular protrusions upon stimulation with insulin (125). Additionally to scanning the surface of a cell, AFM has been used to investigate the surface stiffness of submicron membrane areas of individual cells and map multiple measurements over one cell which has been performed in podocytes by Tandon et al. (126). In this work, the researchers showed that *in vitro* cultured and HIV-infected podocytes were less stiff compared to wild type cells indicating severe cytoskeletal changes of the infected cells. In a work by Wyss and coworkers, AFM has been used to study mechanical properties of whole isolated glomeruli (127). Here, the authors show, that glomerular stiffness was reduced upon application of blebbistatin, an agent inhibiting non-muscle myosin IIa/b as well as cytochalasin D and latrunculin B, both reagents that inhibit actin polymerization. Additionally, they investigated glomerular capillary wall stiffness in Col4a3 knockout mice, which was significantly smaller, compared to wild types highlighting the essential role of the collagen 4 α3 chain in mechanical stabilization of the GBM to withdraw tensile stress along the glomerular capillary wall. In a study of 2018, Embry and coworkers used application of protamine sulfate on cultured podocyte to model mild cytoskeletal changes as seen in MCD, hence showing significant easier plasma membrane indentation quantified by AFM (128).

## Selection of the appropriate method and possible routine application

As shown above, there is a huge variety among the currently used methods in podocyte research, each with its own possibilities and probable pitfalls (Table 1). If one wants to perform 3D imaging of large volumes in native or cleared tissue one should choose SPIM or MPM. Compared with SPIM, MPM offers higher resolution but with, due to the scanning setup, significantly longer imaging time for a sample. For *in vivo* imaging, if high spatial resolution is needed, one should prefer MPM whilst SPIM is the method for imaging highly dynamic processes as demonstrated by imaging of a beating zebrafish heart by Mickoleit et al. (129). Both methods offer relatively easy multichannel imaging and a large selection of appropriate fluorophores.

**Table 1:** Comparison of the different techniques discussed above.

**Technique**	**Typical resolution (xy/z in nm)**	**Used for**	**Preparation**	**Costs**	**Possible diagnostic value**
**VOXEL RESOLUTION**					
Lightsheet/SPIM	200/1,000	Intravital SPIM, embryos, fixed organs	++	++	–
MPM	200/500	Intravital MPM, fixed thick slices	++	++	+
3D-SIM	100/300	Patient biopsies, rodent tissue	+	++	+++
ExM	70/140	Cultured cells, patient biopsies, rodent tissue	+++	+	+++
STED	20/50	Cultured cells, rodent tissue	++	+++	++
dSTORM/PALM	20/50–60	Cultured cells, human and rodent tissue	++	+++	+
SBF-SEM/FIB-SEM	3–30/3–30	Large volume electron microscopy	+++	++++	–
ET/cryoET	4/4	Protein interactions	+++	++++	–
AFM	30/0.1	Measurement of mechanic forces, cell surface	++	+++	–

*The typical resolution provided are derived from experiments in biological samples only*.

If sub-diffraction limit resolution is needed for example for the analysis of podocyte foot processes one would select either a technique of the SR spectrum or an ExM approach. The technique offering least demanding sample preparation but yet lowest increase of resolution is 3D-SIM. However, as 3D-SIM works fast and functions with traditional, rapid sample preparation, standard fluorophores, and as commercially available systems are affordable, it is likely that this technique will find its way into diagnostic routine. However, further research will be needed to investigate whether results obtained from 3D-SIM are able to provide yet unknown insights with therapeutic consequences. Among the SR techniques, localization microscopy based methods offer the highest resolution with the main pitfall that sample preparation is demanding and commercial systems are presently rather expensive. Once established, the least costly methods are of the ExM spectrum offering high resolution without expensive microscopic equipment. It is therefore possible, that once standardized, ExM in general and especially the ExPath approach (104) will be used in routine pathological applications with standard fluorescence light microscopes.

In the field of 3D EM-based methods, two different principles are available. If one wants to investigate intact podocytes *in situ*, FIB-SEM or SBF-SEM will be used as the largest volumes can be imaged. Typically, SBF and FIB-SEM offer similar resolutions (3–30 nm) in the xy-plane, whereas the z-resolution depending on the accuracy of physical milling of the sample is significantly smaller in FIB-SEM (3–30 nm) compared to SBF-SEM (20–50 nm) (130). Most, SBF-SEM studies show reconstruction of larger volumes compared to the studies performed with FIB-SEM. So for volumes >100 μm thickness SBF-SEM would be the method of choice (130). Whilst ET offers the smallest z-volume (<500 nm), it is used for high-detailed imaging of small areas like cell-cell contacts or once combined with vitrification, isolated proteins *in vitro*. Due to the demanding sample preparation, time-consuming image acquisition and data interpretation it is less likely that these techniques will find their way into routine diagnosis of podocytopathies.

In contrast to the above mentioned techniques, AFM works with either direct or indirect interaction with the sample offering the opportunity of the investigation of mechanical properties which is a big issue in podocyte maintenance. However, it is less likely that this technique will find a routine application within the diagnosis of podocytopathies but can be a helpful tool for basic podocyte research.

## Materials and methods

### Multiphoton microscopy (MPM)

Zebrafish larvae of the genotype (Tg(*nphs2*:GAL4); Tg(*UAS*:Eco.nfsb-mCherry); Tg(*fabp10a*:gc-eGFP); mitfa^w2/w2^) at 5 days past fertilization (dpf) were produced and maintained as described before (54). Larvae were anesthetized in Tricaine dissolved in E3 medium and embedded in molten 0.8% agarose in E3 medium. Dual color images where obtained using a Zeiss LSM710MP system coupled to a Coherent Chameleon TiSa laser tuned to 890 and 760 nm for eGFP and mCherry, respectively as described before (53). All experiments were performed in accordance with German animal protection law overseen by the “Landesamt für Landwirtschaft, Lebensmittelsicherheit und Fischerei, Rostock” of the federal state of Mecklenburg—Western Pomerania.

### Three dimensional structured illumination microscopy (3D-SIM)

For SIM, 4 μm paraffin sections where obtained from 4% PFA fixed wild type murine and rat material and mounted on #1.5 high precision coverslips. Sections where dehydrated, antigen retrieved by pressure-cooking in citrate buffer, blocked in 1% FBS, 1% BSA, 1% NGS, 0.1% cold fish gelatin in PBS and co-labeled with guinea pig anti-nephrin (GP-N2, 1:300) and mouse anti-synaptopodin (GP94-C, 1:50, both Progen, Heidelberg, Germany) overnight at 4°C. After extensive washing in PBS, primary antibodies were detected using Cy3-labeled donkey anti-guinea pig and Alexa488-labeled goat anti-mouse F(ab)_2_ fragment (1:600, Jackson Immuno Research, Cambridgeshire, UK) and embedded in Mowiol for microscopy. Image acquisition was performed as described before with additional channel alignment for chromatic aberration using Tetraspek beads embedded in the same mounting medium and Zeiss ZEN black software (8).

### Direct stochastical optical reconstruction microscopy (dSTORM)

WT mouse kidneys were fixed in 4% PFA, embedded in Tissue Tek (Sakura, Staufen, Germany) 1:1 diluted with 1x PBS containing 15% sucrose and snap frozen in liquid nitrogen. One micrometer sections were obtained using a cryostat rotational microtome (Leica Microsystems, Mannheim, Germany) and mounted on ethanol-cleaned #1.5 high precision coverslips. Murine cultured podocytes (131), were washed once in 1x PBS pH 7.4, fixed for 10 min at 37°C with 3% paraformaldehyde and 0.1% glutaraldehyde in 1x PBS. Sections and cells were blocked in 1% FBS, 1% NGS, 1% BSA over night at 4°C and labeled with polyclonal rabbit anti laminin (1:1,000, L9393, Sigma Aldrich, Steinheim, Germany) or monoclonal mouse anti alpha tubulin (1:300, T9026, Sigma Aldrich) at 4°C. Antibodies where detected using Alexa Fluor 647-labeled goat anti-rabbit whole IgG and Alexa Fluor 647-labeled goat anti-mouse IgG fragment antibodies (1:1,000, Jackson ImmunoResearch and Invitrogen, respectively). After extensive washes in PBS, stained samples were postfixed for 10 min at RT. Image acquisition was performed on a Zeiss Elyra PS.1 with slides in a buffer system containing glucose oxidase, catalase and cysteamine. Ten thousand frames where subsequently recorded for each channel. Image reconstruction was performed using Zeiss ZEN 2010 software. Pictures and protocol of Figure 4 are from Siegerist et al. (unpublished micrographs).

## Author contributions

FS performed imaging experiments, analyzed the data and prepared figures. FS, KE, and NE reviewed literature and wrote the manuscript. All authors approved the final version of the manuscript.

### Conflict of interest statement

The authors declare that the research was conducted in the absence of any commercial or financial relationships that could be construed as a potential conflict of interest.
